# Impact of Vitamin D Concentration on Postoperative Outcomes and Complications Following Total Knee Arthroplasty: A Scoping Review of the Literature

**DOI:** 10.3390/nu18111828

**Published:** 2026-06-05

**Authors:** Daniel Jaglarz, Tomasz Strzemecki, Rafał Pankowski, Nina Janowska, Piotr Sypień, Ewa Tramś, Rafał Kamiński, Dariusz Grzelecki

**Affiliations:** 1 Department of Orthopaedics and Trauma Surgery, Ceynowa Hospital in Wejherowo, Jagalskiego 10, 84-200 Wejherowo, Poland; jaglarzd@gmail.com (D.J.); tomek.strzemecki@gmail.com (T.S.); janowskanina@gmail.com (N.J.); 2 Department of Orthopedics, Traumatology and Spine Surgery, Medical University of Gdańsk, Gdańsk Hospital, Nowe Ogrody 1-6, 80-803 Gdansk, Poland; rafalpankowski@wp.pl; 3 Department of Trauma and Orthopedic Surgery, Sebastian Petrycy Healtcare Facility, 33-200 Dabrowa Tarnowska, Poland; piotrsypien@gmail.com; 4 Department of Orthopedics and Musculoskeletal Trauma, Centre of Postgraduate Medical Education, Adam Gruca Orthopedic and Trauma Teaching Hospital, Konarskiego 13, 05-400 Otwock, Poland; ewatrams@gmail.com (E.T.); raf.kaminski@gmail.com (R.K.); 5 Department of Orthopedics and Reconstructive Surgery, Centre of Postgraduate Medical Education, Adam Gruca Orthopedic and Trauma Teaching Hospital, Konarskiego 13, 05-400 Otwock, Poland

**Keywords:** Vitamin D, total knee arthroplasty, PROMs, periprosthetic joint infections

## Abstract

**Background/Objectives:** Despite its beneficial effect on the healing of bone fractures and in the treatment of osteoporosis, there is still a lack of evidence on the impact of clinical outcomes after a total joint arthroplasty (TJA). This review aims to establish the role of vitamin D in clinical outcomes after a total knee arthroplasty (TKA). **Methods:** In this review, PubMed, Scopus and Web of Science databases were cross-checked by two reviewers independently. The inclusion criteria were original human studies published in English from 2014 to 2024. For identification-relevant studies, the search terms used were as follows: “Vitamin D” and “total knee arthroplasty” or “total knee replacement” or “total joint arthroplasty” or “total joint replacement”. Case reports, letters and expert consensuses were excluded from the analysis. Finally, 19 studies were included in this review. **Results:** A literature review shows that vitamin D may have an impact on patients treated for osteoarthritis (OA) of the knee with a significant prevalence of hypovitaminosis in orthopedic patients. The influence was observed for periprosthetic joint infections: PJI patients have significantly lower vitamin D levels than primary ones. Also, a greater incidence of revision knee surgery due to PJIs in the deficient group compared to the non-deficient group at a one-year follow-up was found, of up to a 2-fold increase. This affects the clinical outcome with a lower Knee Society Score (KSS) functional score in the vitamin D-deficient group. **Conclusions:** The current data suggest that the vitamin D metabolism pathway and its implications in orthopedic patients, especially those treated with TKA surgery, may be a significant factor that improves clinical and functional outcomes. A possible relation between a low preoperative concentration of vitamin D and its impact on the outcomes, such as the length of the hospital stay, implant survival, and risk of complications, is needed to support these findings in multicenter, prospective studies and randomized controlled trials.

## 1. Introduction

Although total knee arthroplasty (TKA) is an established and effective treatment for advanced osteoarthritis (OA), approximately 10% of patients remain dissatisfied after technically well-performed primary procedures [1]. Therefore, identifying perioperative factors that influence patient satisfaction is essential for enhancing clinical and functional outcomes. Recent research has primarily focused on factors related to surgical precision and accuracy, which may be improved through various methods such as the surgical approach, patient-specific instrumentation, and robotic assistance [2]. In recent years, a growing concept of personalized implant alignment (kinematic and functional) has been noted; however, the current literature is insufficient to support these strategies regarding clinical outcomes in long-term follow-up.

Several other studies have analyzed patient-related factors, including the age, sex, body mass index (BMI), level of physical activity, severity of OA, and presence of various comorbidities [3,4]. It was confirmed that the postoperative outcomes and the occurrence of potential complications are directly linked to patient dissatisfaction following a TKA [5]. According to data from large national registries (Swedish, United States, Australian, and British), a periprosthetic joint infection (PJI) is currently the most common cause of failure after a TKA, followed by aseptic loosening and instability [6,7].

**Figure 1:**
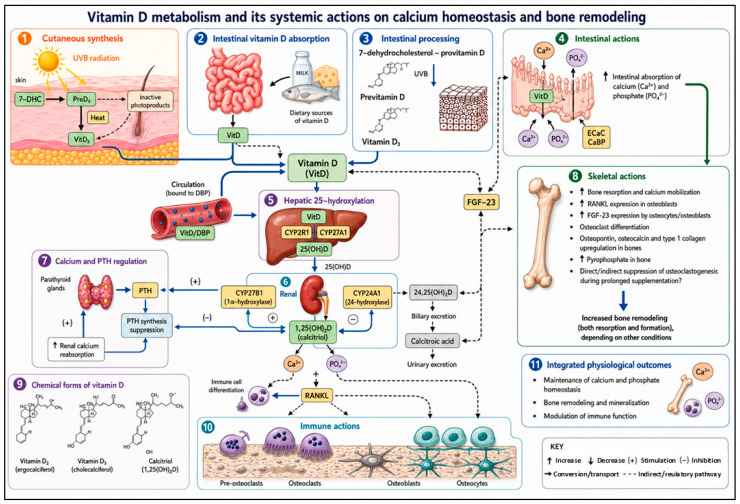
Vitamin D metabolism and its systemic actions on calcium homeostasis and bone remodeling. 7-DHC—7-dehydrocholesterol; CY—cytochrome; DBP—vitamin D-binding protein; ECaC—epithelial Ca(2^+^) channels; FBF—fibroblast growth factor; PTH—parathormone; RANKL—receptor activator for nuclear factor κ B ligand; UVB—ultraviolet B; VitD—vitamin D.

Among the various patient-related factors that may influence postoperative outcomes, implant survival, and the risk of complications following a TKA, the perioperative concentration of vitamin D in blood has attracted the interest of researchers; however, it remains insufficiently investigated. Vitamin D is a steroid hormone essential for proper bone metabolism in humans. Vitamin D is essential for the proper functioning of the skeletal system, as it aids in the absorption of calcium and phosphorus from the gastrointestinal tract. Its deficiency can lead to osteoporosis, osteomalacia in adults, rickets in children, and an increased risk of fractures. Additionally, studies suggest that vitamin D may play a role in immune system function, which could be significant in preventing autoimmune diseases, such as multiple sclerosis, and in reducing the risk of developing certain cancers and cardiovascular diseases, even infections and OA [8]. Recent evidence indicates that OA is not merely a degenerative cartilage disorder, but a complex whole-joint disease involving interactions among chondrocytes, osteoblasts, osteoclasts, synovial cells, immune cells, inflammatory cytokines, and extracellular matrix-degrading enzymes [9,10]. Vitamin D may influence several of these pathways through its immunomodulatory and anti-inflammatory effects. Vitamin D receptors are expressed in chondrocytes, osteoblasts, and synovial fibroblasts, suggesting a direct role in cartilage metabolism and bone remodeling. Experimental studies have demonstrated that vitamin D can modulate the expression of inflammatory mediators such as IL-1β, IL-6, TNF-α, and matrix metalloproteinases, which are involved in cartilage degradation and OA progression [9,10]. Furthermore, vitamin D may contribute to maintaining subchondral bone integrity, skeletal muscle strength, and postoperative functional recovery following a TKA [9,10]. Studies show that vitamin D plays an important role in bone metabolism, but its effect on bone healing is uncertain [11]. Nevertheless, in spine surgery, deficiency is suspected to be an independent factor of nonunion risk [12]. In TKA patients, vitamin D stimulates osteointegration and the remodeling process [13]. Its effect on the immune system leads to a reduction in inflammatory responses and a lower infection risk [14]. Vitamin D alters muscle strength and improves proprioception as well as has a beneficial effect on mental status via serotonergic and dopaminergic systems that can help in the rehabilitation process, faster recovery and better clinical outcomes [15]. A schematic figure regarding the mechanism of action on bone healing and the metabolism of vitamin D is presented in Figure 1.

This review aims to analyze the influence of the vitamin D concentration in patients who underwent a TKA and its impact on the postoperative results and complication rate.

## 2. Materials and Methods

**Figure 2:**
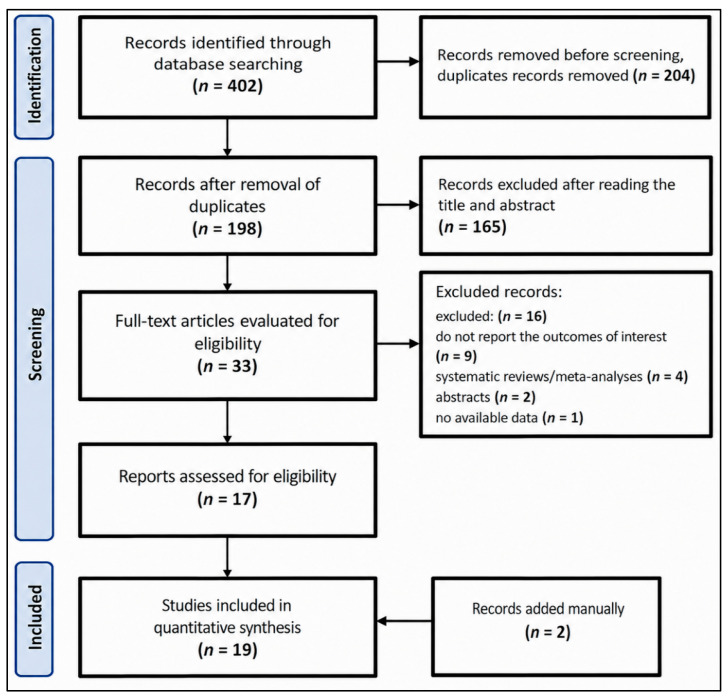
Flow diagram of the articles extracted from the electronic databases following the key terms.

This study received the agreement of the Bioethics Committee at the District Medical Chamber in Gdansk (no. KB—4/26) and was conducted according to the 1964 Declaration of Helsinki. In this scoping review that examined the vitamin D concentrations in patients who underwent a TKA and its impact on postoperative outcomes and complications, the PubMed and Scopus databases were independently searched by two reviewers. Only original human studies published in English from 2014 to 2024 were included. For identification-relevant studies, the search terms used were as follows: “Vitamin D” and “total knee arthroplasty” or “total knee replacement” or “total joint arthroplasty” or “total joint replacement”. Case reports, letters, expert consensus statements, studies involving total joint arthroplasties (TJAs) other than the knee, and revision TKAs were excluded from further analysis in this review. Finally, 402 studies were identified. After the exclusion of duplicates and records that did not fit the selection criteria, 19 original studies were included in the review. A flow-chart is presented in Figure 2.

For each study, depending on its design, demographic data (including sex and age [mean or median with range]) and clinical data (such as the vitamin D threshold and concentration, outcomes and/or implant survival rate, clinical and functional assessment scales, complications, and key findings) were extracted and are summarized in Table 1 and Table 2.

## 3. Results

A total of 19 studies were included in this scoping review [14,15,16,17,18,19,20,21,22,23,24,25,26,27,28,29,30,31,32]. There were nine studies assigned to the clinical and functional outcomes section. Eleven papers presented complications. Two papers were put in both groups because they regarded both sections.

### 3.1. Clinical and Functional Outcomes

A total of nine studies comprising the clinical and functional outcomes of TKAs in relation to the vitamin D concentration were included in the final analysis [16,17,18,19,20,21,22,23,24]. The cohorts ranged from 87 to 176 patients, with the proportion of females in the deficient group varying between 51% and as high as 90.7%. The range of hypovitaminosis varied from less than 12 ng/mL to less than 30 ng/mL. The threshold for sufficiency was set from 12 ng/mL or higher up to greater than 40 ng/mL. The percentage of vitamin D deficiency in the analyzed cohorts ranged from 23.74% to 80%. A higher prevalence of vitamin D deficiency was noted among females, depending on the cut-off values used in the respective studies (Table 1). In six out of the nine studies included in this review section, a negative effect of vitamin D deficiency on the postoperative outcomes in Knee Society Score (KSS) and Western Ontario and McMaster Universities Osteoarthritis Index (WOMAC) was observed.

### 3.2. Complications

In 12 studies, the impact of vitamin D on the complication occurrence was analyzed [20,21,25,26,27,28,29,30,31,32,33,34]. The investigated cohorts ranged from 109 to 142,147 participants, and females constituted a percentage range from 51% to 80%. The thresholds of insufficiency were set from <20 ng/mL to <30 ng/mL. The hypovitaminosis percentage ranged from 13,2% to even 100%. A negative impact of hypovitaminosis was identified in 10 out of the 12 studies analyzed. This refers to a higher complication rate, mentioned in six out of 12 papers, such as: a surgical site infection (SSI), an infection requiring explantation of implants, stiffness, a PJI, cellulitis, a wound infection and other general complications, for example, deep vein thrombosis (DVT) [20,27,28,31,32,33]. It has also been established that patients with a PJI and those scheduled for primary or revision artroplasty have significantly lower serum levels of vitamin D [21,26,29,30,34]. Two studies found no association between the vitamin D levels and the risk of aseptic loosening in patients undergoing a primary TKA [21,34], while only one study reported no significant difference in the periprosthetic fracture risk when comparing vitamin D-deficient and -sufficient groups [34] (Table 2).

## 4. Discussion

An increasing number of studies indicate the negative impact of low vitamin D levels on surgical outcomes and recovery. Studies by Jansen et al. and Shin et al. suggest that low vitamin D levels are associated with longer hospital stays, an increased number of postoperative complications such as infections, and poorer functional outcomes after surgery [16,20]. Vitamin D-deficient patients had a markedly longer stay by +1.0 day (95% CI: 0.2 to 1.6 days); *p* = 0.03. The WOMAC score was also significant (*p* = 0.04) and indicated a worse functional outcome also at the long-term follow-up after eight years (WOMAC: +5.0, range: 0.2–9.8) [16]. Early postoperative functional outcomes following a TKA appear to be adversely affected by vitamin D deficiency in terms of the functional part of KSS [20]. In a study by Shin et al., the authors found a statistically significant difference detected on the KSS functional part between the sufficient and insufficient groups postoperatively, with a lower score in deficient patients [20]. The mean postoperative functional KSS was significantly less in the vitamin D-deficient group (serum 25-hydroxyvitamin D3 (25(OH)D) levels < 12 ng/mL) than in the vitamin D-non-deficient group (serum 25(OH)D levels ≥ 12 ng/mL) (67.2 vs. 73.4, *p* = 0.031). This may be explained by a range of vitamin D effects on muscle function. Most authors define hypovitaminosis D as serum 25(OH)D levels below 20 ng/mL (50 nmol/L), while others use a threshold of below 30 ng/mL (75 nmol/L). Consequently, patients with serum concentrations ≥30 ng/mL were generally considered to have a sufficient vitamin D status. This variability in diagnostic thresholds may partially explain the heterogeneity of the reported outcomes and the lack of a unified consensus regarding the influence of vitamin D on the TKA outcomes [35].

The study by Hegde et al. further indicates that patients with a vitamin D deficiency have a higher risk of postoperative complications, underscoring the need for correcting vitamin D deficiencies before surgery to improve outcomes [33]. A 25D deficiency is associated with a higher risk of postoperative complications, including a SSI requiring irrigation and debridement (OR: 1.76; *p* = 0.001) and explantation of the prosthesis (OR: 2.97; *p* < 0.001), stiffness requiring manipulation under anesthesia (OR: 1.69; *p* < 0.001), DVT (OR: 1.80; *p* < 0.001), and cardiocerebrovascular events (OR: 1.73; *p* = 0.006) [33].

A vitamin D deficiency is consistently associated with poorer functional outcomes, higher rates of revision and infection, and longer hospital stays after a TKA, as measured with WOMAC, KSS, and AKSS scores [25]. A study by Mainar et al. was conducted on 120 TKAs divided into sufficient (>30 ng/mL) and insufficient (<30 ng/mL) groups that comprised 64 and 56 individuals [21]. All patients were supplemented postoperatively from day 14 with oral vitamin D for 4 weeks. It showed a significant difference in only the preoperative WOMAC score between the two groups. Postoperatively, a comparison of the WOMAC score, FS-12, KS-KSS, and FS-KSS in sufficient and insufficient groups of patients showed no significance in terms of a statistical analysis showing comparable results in both groups [21].

Hegde et al. demonstrated, in patients with a vitamin D deficiency, that there is a 66% increase in postoperative stiffness requiring manipulation under anesthesia at 3 months and a 69% increase at 1 year (*p* < 0.001) [33]. The study by Jensen et al. showed a lower KSS score in the vitamin D-deficient group [17]. The difference was statistically significant preoperatively, which makes these studies worthy of interest. The postoperative KSS scores were similar between the sufficient and insufficient groups, with no statistical significance. Nevertheless, the mean postoperative KSS at six months was also lower in the vitamin D-deficient group (74.6 vs. 80.4), but this difference was not statistically significant (*p* = 0.075). It has to be mentioned that the postoperative deficient cohort was only 23 patients and there were 79 individuals from the sufficient group. This could alter the perspective in terms of underestimation.

In an interesting paper by Morrison et al., the investigators examined peri-operative vitamin D supplementation in TKA patients: it influences the inflammatory response measured with the IL-6 and IL-10 biomarker ratio, reducing it with no impact on the WOMAC score or fall rates [36]. Hwang et al. demonstrated an analysis on 1013 knees treated with a TKA in a one-year follow-up; there were no significant differences between the sufficient and insufficient groups of patients [22]. There was a large number of hypovitaminosis D cases, at nine < 20 ng/mL, which was 80% of the participants. The clinical differences were measured using the VAS, KSS and WOMAC scores. A preoperative vitamin D deficiency (<20 ng/mL) was highly prevalent, but did not substantially affect the short-term postoperative functional outcomes (VAS, KSKS, KSFS, WOMAC) in elderly women one year after a TKA [22]. To emphasize, Weintraub et al. investigated supplementation with 50,000 international units of oral vitamin D3 on the day of surgery [19]. They failed to demonstrate statistically significant differences in the functional KSS, TUGT times, or complications in the early postoperative period compared to the placebo. No differences were found in the KSS, TUGT, or complication rates between the vitamin D3 and placebo groups [19].

Kahn et al. divided patients into two groups depending on their vitamin D serum level before the surgery, with the cut-off set at 30 ng/mL [24]. At 3 months postoperatively, the functional KSS showed a significant difference between the two groups (65.98 ± 5.10 in the insufficient group and 74.87 ± 5.02 in the sufficient group, *p* < 0.01). The performance tests showed significant differences between the two groups (16.46 ± 2.78 vs. 15.12 ± 3.37, *p* = 0.02 for AST; 8.48 ± 2.06 vs. 7.49 ± 1.88, *p* = 0.01, for SWT). This means that the vitamin D levels significantly affected the postoperative functional outcome in TKA patients [24]. To comment on the PROM (patient-reported outcome measures) evaluation in terms of the vitamin D 25OH status, it has to be mentioned that these studies used different cut-off levels for sufficiency and insufficiency ranging from 12 ng/mL to 50 ng/mL. This makes it hard to compare them. Further studies are needed to determine the real impact on Patient-Reported Outcome Measures (PROMs). Lee et al. demonstrated that hypovitaminosis D is likely to be a new risk factor for the development of moderate-to-severe persistent pain after knee arthroplasty [23]. The analysis suggested a 2- to 3-fold increased risk of moderate-to-severe persistent pain with hypovitaminosis D [23]. The study did show a high prevalence of preoperative hypovitaminosis D. Moderate-to-severe hypovitaminosis D had subtle effects on pain intensity scores in the early postoperative period, but the health-related quality of life outcomes at 3 months were comparable with other patients with a sufficient level of vitamin D concentration.

The current literature is lacking big data investigating an association of low vitamin D serum levels and complications after a TKA surgery. First of all, vitamin D plays an important role in the immune system by binding to its vitamin D receptor and stimulating the transcription of antimicrobial peptides, for example, defensin and cathelicidin [32]. In the orthopedic field, not only do general infections play a role, such as pneumonia, urinary tract infections, and sepsis, but wound-healing problems, SSIs and PJIs also play a role. As mentioned in the introduction, PJIs are the most devastating complication in TKAs, and are the first most common cause of revision surgery [32].

Maier et al. conducted a study of 109 patients who underwent a total joint replacement surgery: TKAs, THAs and total shoulder arthroplasties [32]. The authors found that all the patients presenting with a PJI or aseptic loosening of the implant showed low serum vitamin D levels. There was no difference in the vitamin D levels comparing patients with a primary prosthesis and those with an aseptic loosening one. A statistically significant difference was found between a primary arthroplasty and those with a PJI, with *p* < 0.001. In addition, the same significance was found between the aseptic loosening groups and the PJI group, with *p* < 0.001 [32]. In conclusion, Maier proved a high incidence of hypovitaminosis D in patients scheduled for joint arthroplasties, with a severe deficiency for those with a PJI (13.9 ng/mL ± 6.54). Zajonz et al. analyzed the prevalence of reduced calcium and vitamin D serum levels in three groups of patients. [29]. The first was a control PJI group for which the International Consensus Meeting on Periprosthetic Joint Infection criteria were used (major and minor ones). Second, there was a group of primary operations, and the third one was the aseptic loosening group. The major finding of this study was that the acute PJI patients had significantly lower levels of calcium and vitamin D than both other groups. An additional finding showed a lower level of protein and albumin in the PJI group, for both chronic and acute patients. According to the risk of a periprosthetic fracture after a TKA, only one study conducted by Pilc et al. demonstrated a lower risk of a periprosthetic fracture in patients with a vitamin D deficiency than in patients with a sufficient level (0.3% vs. 0.5%, respectively); however, this was not statistically significant (*p* = 0.22) [34]. In addition to the preoperative vitamin D status, other preoperative factors may influence the risk of complications and should also be taken into consideration [37]. These are the patient’s overall nutritional status, comorbidities, inflammatory response, type and duration of surgical procedure, anesthesia-related factors, postoperative mobilization, and infection control measures. Furthermore, variables such as the age, body mass index, smoking status and use of medications may affect the complications following a TKA [38,39].

An interesting paper from Birinci et al. suggests that oral supplementation of vitamin D before the surgery may play a role [27]. The authors performed their study on two groups of patients, one that received 300,000 IU of vitamin D 2 weeks before the surgery (deficient patients with serum vitamin D levels < 30 ng/dL) and a control group that did not receive the supplementation treatment. The groups were compared 90 days after surgery. This revealed that the total number of complications, superficial wound infections and peri-prosthetic cellulitis were significantly higher in the group that did not receive supplementary treatment. The mortality, readmission rate and PJIs were similar between the groups [27].

In a study by Hedge et al. that investigated 6593 TKAs, 868 were vitamin D-deficient [33]. The interesting and concerning result of this paper is the finding of a greater incidence of revision knee surgery due to PJIs in the deficient group compared to the non-deficient group at the one-year follow-up (OR, 2.97; 95% CI, 2.04–4.31; *p* < 0.001). Importantly, this suggests that insufficient patients have a 2-fold increased PJI risk in 1 year, which is similar to big national register data in which PJIs are the most common reason for revision surgery. The data mentioned above suggest that great care must be taken to further investigate this topic to establish the effect of hypovitaminosis D on the complication rates. In particular, the effect on PJIs should be set, as it is the most devastating complication that requires another surgery with unsatisfactory results. Hedge et al. also demonstrated more frequent postoperative complications such as DVT, myocardial infarctions and cerebrovascular accidents.

These papers indicate a potential role of vitamin D homeostasis and supplementation in TKA patients. This means that addressing vitamin D may reduce early complications. In a big study by Kong et al., which included 142,147 patients, the authors admitted that the implant survival was significantly improved after supplementation of calcium and vitamin D for 1 year before the surgery [28]. It had an impact on reducing the risk for revision surgery after a TKA due to implant loosening and PJIs. The implant survival at the 5-year follow-up increased from 98.42% to 99.48% in all subjects and from 75.25% to 93.30% in subjects with infections. This shows a significant reduction in the impact failure rate by 67.1% and 72.9% [28]. In conclusion of this study, it is important that supplementation of calcium and vitamin D (800 IU or greater) for 1 year before the surgery adversely reduces the complication rate of aseptic loosening and infections.

In terms of this finding, Arshi et al. prepared a cost-effectiveness model of vitamin D repletion in TKAs [26]. They proved that a repletion of vitamin D is a cost-effective mechanism of reducing the risk of PJIs. Nonselective repletion is more effective than selective repletion in terms of expensive laboratory serum level testing.

Additional studies, such as those conducted by Liu et al. and Mouli et al., have demonstrated that preoperative vitamin D supplementation can effectively correct hypovitaminosis D and potentially improve the surgical outcomes [25,30]. The study by Weintraub et al. was one of the first randomized controlled trials to confirm that vitamin D3 supplementation before surgery can reduce the incidence of postoperative complications and accelerate patient recovery, but there was no statistical significance [19].

These studies, along with findings from other research, such as those by Kong et al., which analyzed the relationship between vitamin D supplementation and implant survival, and by Arshi et al., which presented a cost-effectiveness model for vitamin D repletion before a TKA, highlight the importance of assessing and optimizing vitamin D levels in patients preparing for a TKA surgery [26,28]. The high prevalence of vitamin D deficiency in this patient population underscores the need for routine monitoring and supplementation, which could help reduce the risk of complications, improve joint function post-surgery, and enhance the overall quality of life for patients after the procedure. At the end, Jansen et al. examined 138 patients who underwent a TKA. The patient cohort was divided into sufficient (median: 26 ng/mL, range: 16.4–70.8 ng/mL) and insufficient (median: 32, range: 2.4–16 ng/mL) vitamin D groups. This study indicated a difference in the hospital stay of +1 day, range: 0.2–1.6 days in the deficient group, with statistical significance [16]. In Birinci et al.’s paper, it is noted that there is an insignificant relation between low vitamin D levels and a prolonged hospital stay in revision procedures [27].

This review paper aimed to identify the vitamin D influence on epidemiology, clinical outcomes and complications related to TKA surgery. A TKA is a procedure that is growing in numbers and is expected to increase by 673% to 2030 [40]. Unfortunately, there are still about 10% of patients dissatisfied with the procedure [41,42]. Patients undergoing a TKA are deficient or insufficient in their serum vitamin D levels, with cut-offs set at <30 ng/mL and <20 ng/mL [43]. However, the definition of hypovitaminosis D is not standardized across the literature, which limits direct comparability between studies and may affect the interpretation of the reported associations. Nevertheless, most included studies applied thresholds close to 20 ng/mL. According to the 2023 guidelines for vitamin D supplementation, serum 25(OH)D concentrations ≤ 20 ng/mL are considered indicative of a vitamin D deficiency and concentrations between 20 and 30 ng/mL reflect a suboptimal vitamin D status, whereas concentrations between 30 and 50 ng/mL are regarded as adequate to optimal vitamin D levels [35]. The prevalence of hypovitaminosis appears to be even higher in patients scheduled for revision procedures, reaching approximately 55% [40]. Similar prevalence rates were reported by Forrest et al. in the general US population, where hypovitaminosis D affected nearly 42% of individuals [41].

The association between the PROMs and vitamin D serum levels has been established in several trials, with the most popular being WOMAC and KSS score evaluation. The effect of hypovitaminosis D remains unclear in terms of different cut-offs for diagnosis and makes trials hard to compare. However, it is quite certain that there is an influence on PROMs, especially in patients with levels below 20 nq/mL, with statistically proven significance. Further investigation on this topic is needed to establish cut-offs for diagnosis and standardized questionnaires. The available literature indicates that assessment and correction of a vitamin D deficiency prior to an arthroplasty may be clinically beneficial. Although no standardized supplementation protocol has been established, vitamin D3 supplementation at doses of approximately 800–2000 IU/day in cases of insufficiency and 2000–4000 IU/day in patients with a confirmed deficiency, administered for at least 6–8 weeks preoperatively, appears to be a reasonable strategy aimed at achieving serum 25(OH)D concentrations above 30 ng/mL. Further prospective randomized studies are required to determine optimal supplementation regimens and clarify the role of vitamin D optimization in patients undergoing a TKA [44].

According to the 2023 Polish guidelines, adults with a vitamin D deficiency may require therapeutic supplementation doses of approximately 4000 IU/day, with the reassessment of serum 25(OH)D concentrations after 8–12 weeks of treatment. The guidelines additionally recommend re-evaluation of the vitamin D status after 1–3 months of cholecalciferol therapy [35].

In connection with the PROMs, it is very important in orthopedic patients that have undergone a knee replacement surgery to have no complications after surgery. The most devastating complication, and also the most common one in the early and late period, is a PJI. As we know from the basic science, vitamin D is an important factor that influences the immune system. There seems to be some evidence that suggests an association between a low vitamin D status and revision knee surgery due not only to a PJI, but also to aseptic loosening. Vitamin D-deficient patients are more likely to undergo a revision surgery. It is worthwhile to detect these patients early. It would be optimal to detect them before the primary surgery in terms of improving the clinical outcome and reducing the complication rate, especially reducing the revision rate, which is a very dangerous procedure and also expensive for health providers. If hypovitaminosis is diagnosed, patients could be delayed from surgery or put into supplementation therapy to be operated on under optimal clinical status with no hypovitaminosis. The included studies differed in their definitions of vitamin D deficiency and insufficiency, which may limit direct comparability between studies. In addition, no formal quality assessment of the included studies was performed, and the methodological quality of the analyzed studies may have varied. Furthermore, some studies did not demonstrate significant associations between the vitamin D status and the clinical outcomes.

## 5. Conclusions

Our review shows the importance of the perioperative vitamin D concentration in individuals undergoing a primary TKA. The rate of hypovitaminosis is of a high prevalence. Hypovitaminosis D seems to have a negative impact on the outcomes after a primary TKA. It is worthwhile to detect patients scheduled for a TKA early in terms of their vitamin D level to introduce treatment. It is a cheap and easy way to avoid complications. Vitamin D has a multifocal influence on musculoskeletal health, especially in orthopedic patients. This influence is mainly connected with vitamin D deficiency, which may lead to the development of postoperative complications, such as low PROMs, an increased risk of infections, wound healing complications, or even mortality.

The available evidence suggests a potential association; however, the findings remain partially inconsistent across studies.

## Figures and Tables

**Table 1:** Summary of studies analyzing clinical and functional outcomes following TKA in relation to blood vitamin D concentration.

Author	Country	Study Group Size	Males/Females	Age (Mean or Median with Range) [Years]	Vitamin D Threshold (ng/mL)	% of Patients with Vitamin D Deficiency	Clinical and Functional Scales	Outcomes/Implant Survival	Key Findings
Jansen et al. [16]	United Kingdom	138	Deficient group: female, 20; male, 13 Non-deficient group: female, 61; male, 44	Deficient group: 69.9 years Non-deficient group: 72.8 years	≤16 ng/mL	33 out of 138 (~24%)	Length of hospital stay: significantly longer stay by +1.0 day (95% CI: 0.2 to 1.6 days); *p* = 0.03. WOMAC: significant (*p* = 0.04) worse functional outcome at long-term follow-up after eight years (WOMAC: +5.0, range: 0.2–9.8).	WOMAC; length of hospital stay.	Preoperative vitamin D deficiency was associated with prolonged hospital stay and worse long-term functional outcomes as measured by WOMAC scores.
Jansen et al. [17]	United Kingdom	139	Male: 58 Female: 81	71.4 (mean), range of 48–88	Deficiency: <16 ng/mL	33 out of 139 (~23.74%)	The mean preoperative KSS was lower in the vitamin D-deficient group than in the sufficient group (31.5 vs. 37.1, *p* = 0.047). The mean postoperative KSS at six months was also lower in the vitamin D-deficient group (74.6 vs. 80.4), but this difference was not statistically significant (*p* = 0.075).	KSS.	Lower KSS score in vitamin D-deficient group.
Morrison et al. [18]	United Kingdom	413	N/A	N/A	N/A	N/A	Biomarker response: statistically significant reduction in IL6:IL10 ratio at 24 and 48 h post-op with supplementation. Functional outcomes: no significant improvement in WOMAC scores or falls rate.	WOMAC (self-reported knee function); falls incidence.	Perioperative vitamin D supplementation in TKA patients reduced inflammatory biomarkers (IL6:IL10 ratio), but had no measurable effect on functional outcomes such as the WOMAC scores or postoperative fall rates; evidence quality was limited and heterogeneous.
Weintraub et al. [19]	USA	107 Placebo group: 50 Vitamin D3 group: 57	Placebo group: male, 23; female, 27 Vitamin D3 group: male, 28; female, 29	Placebo group: 64.5 Vitamin D3 group: 63.7	N/A	N/A	No difference in improvement of KSS and Timed Up and Go Test (TUGT) at 3 weeks or 6 weeks from baseline. Four complications in the placebo cohort within the first 90 days post-operatively. Five complications in the vitamin D3 cohort (P 1⁄4 1.0).	KSS; TUGT.	Supplementation with 50,000 international units of vitamin D3 on the day of surgery failed to demonstrate significant differences in functional KSS, TUGT, or complications in the early postoperative period compared to placebo. No differences were found in KSS, TUGT, or complication rates.
Shin et al. [20]	South Korea	92 (87 performed all required assessments)	Deficient: male, 4; female, 39 Non-deficient: male, 5; female, 39	Deficient: 70.7 ± 6.8 Non-deficient: 72.4 ± 4.2	Adequate levels, 20 ng/mL; deficiency, <12 ng/mL; insufficiency, 12–20 ng/mL Deficient group, serum 25(OH)D levels: <12 ng/mL; non-deficient group, serum 25(OH)D levels: ≥12 ng/mL	49.43% (43 out of 87)	Vitamin D-deficient group had significantly poorer postoperative outcomes as assessed by functional KSS, AST, and SMT. Preoperative KSS (31.5 vs. 37.1, *p* = 0.047) when compared to patients without deficiency.	KSS, AST, SMT, STS, TUGT.	Early postoperative functional outcomes following TKA appear to be adversely affected by vitamin D deficiency.
Maniar et al. [21]	India	120	120	Deficient: male, 14; female, 50 Sufficient: male, 9; female, 47	Deficient: ~67 Sufficient: ~69	Deficient, <30 ng/mL; sufficient, ≥30 ng/mL	53.3% (64 out of 120)	Preoperative function was lower in osteoarthritic patients with vitamin D deficiency (WOMAC score; *p* = 0.040), but at 3 months, all functional scores were similar.	WOMAC (pain, stiffness, physical function); SF-36 (general health status); KSS.
Hwang et al. [22]	South Korea	704 patients (1013 knees), with a subgroup of 220 patients (220 knees)	704 patients (1013 knees), subgroup of 220 patients (220 knees)	Male: 76 Female: 628 Subgroup (functional analysis): 220 females only	Non-def: 69.6 (55.0–84.0) Deficient: 67.4 (46.0–86.0)	Deficiency, <20 ng/mL; sufficient, ≥20 ng/mL	80% (176 out of 220)	The number of vitamin D-deficient patients was 556 (79.0%). The vitamin D level was negatively correlated with weight only (*p* = 0.033). No significant differences were observed between the groups in terms of the postoperative VAS score, KSKS, KSFS, and WOMAC score.	VAS; KSKS; KSFS; WOMAC.
Lee et al. [23]	China	191 Patients undergoing TKA	Male: 153 Female: 38	68 (64–72)	Deficiency: severe, <5 ng/mL; moderate, 5–11.6 ng/mL; mild, 12–19.6 ng/mL; sufficient, 20–88 ng/mL	7.5–36.4%	There was a vitamin D status–time interaction on EQ-5D VAS (*p* = 0.02), but not for the EQ-5D index. There was no effect of vitamin D status on the total WOMAC index, WOMAC pain, and stiffness subscales. The effect of vitamin D status groups on WOMAC function was marginally significant (*p* = 0.07); preoperative WOMAC function was lowest in the moderate-to-severe hypovitaminosis D group compared with the sufficient group (MD: 13.1, 95% CI: 24.1 to 2.0). Preoperative prevalence of mild and moderate-to-severe hypovitaminosis D was 36.4% (95% CI: 30.0–43.3%) and 7.5% (95% CI: 4.3–11.9%).	EQ-5D VAS; WOMAC.	Moderate-to-severe hypovitaminosis D was associated with a transient higher pain intensity at rest scores without affecting the total morphine consumption or the quality of recovery after surgery. The regression analysis suggested a 2- to 3-fold increased risk of moderate-to-severe persistent pain with hypovitaminosis D. Hypovitaminosis D is likely to be a new risk factor for the development of moderate-to-severe persistent pain after a knee arthroplasty.
Khan et al. [24]	Pakistan	110	Male: 48 Female: 62	Deficient: 60.87 ± 5.10 Sufficient: 60.09 ± 4.78	Deficient: <30 ng/mL; sufficient: ≥30 ng/mL	50% (55 out of 110)	Three months postoperatively: patients with preoperative vitamin D deficiency had significantly lower KSS scores (65.98 ± 5.10 vs. 74.87 ± 5.02; *p* < 0.01), slower AST times (16.46 ± 2.78 s vs. 15.12 ± 3.37 s; *p* = 0.02), and slower SWT times (8.48 ± 2.06 s vs. 7.49 ± 1.88 s; *p* = 0.01) compared to vitamin D-sufficient patients.	KSS; AST; SWT.	Preoperative vitamin D deficiency was associated with poorer postoperative functional outcomes in TKA patients, highlighting the importance of assessing and correcting vitamin D levels before surgery.

**Table 2:** Summary of studies analyzing postoperative complications following TKA in relation to blood vitamin D concentration.

Authors	Country	Study Group Size	Males/Females	Age (Mean or Median)	Vitamin D Threshold (ng/mL)	% of Patients with Vitamin D Deficiency	Complications	Key Findings
Shin et al. [20]	USA	6593	Deficient: male, 197; female, 671 Sufficient: male, 1402; female, 4323	N/A	Deficiency defined as <20 ng/mL; sufficient level defined as ≥20 ng/mL	13.2% (868 out of 6.593 patients)	Tibial/femoral revision (at 3 months) deficient: 4 (0.46%); sufficient: 17 (0.30%). Tibial/femoral revision (at 1 year) deficient: 12 (1.38%); sufficient: 57 (1.00%). Explantation of prosthesis (at 1 year) deficient: 13 (1.50%); sufficient: 26 (0.45%).	A 25D deficiency is associated with a higher risk of postoperative complications: SSI requiring irrigation and debridement along with implant explantation, stiffness requiring manipulation under anesthesia, DVT, cardiocerebrovascular events.
Maniar et al. [21]	Germany	109	Male: 47 Female: 62	65 (±9.2)	Deficiency: <20 ng/mL Insufficient: 20–30 ng/mL Sufficient: >30 ng/mL	64% (70 out of 109)	Second surgery due to (*n* = 70): prosthesis loosening, 16 (52%); PJI, 43 (86%).	No significant differences between patients scheduled for a primary TJA and patients with aseptic loosening (*p* = 0.58). Significant differences in 25OHD levels were found between patients with PJI and patients scheduled for a primary TJA (*p* < 0.001).
Mouli et al. [25]	USA	174 patients	Male: 79 Female: 95	65.33 (±8.59)	Deficiency: <30 ng/mL	100%	Significantly longer length of stay after elective arthroplasty hypovitaminosis D has been linked to higher risk of 90-day complications and PJI requiring revision surgery.	High-dose vitamin D supplementation (50,000 IU weekly) effectively corrected hypovitaminosis D in patients awaiting a TKA. Most patients reached sufficient vitamin D levels within 4–6 weeks prior to surgery. Preoperative correction of vitamin D deficiency is a simple, low-cost, and low-risk intervention that may contribute to improved postoperative outcomes.
Arshi et al. [26]	USA	Cost-effectiveness simulation model based on a cohort of 10,000 TKA patients			Deficiency: <20 ng/mL		PJI.	Preoperative vitamin D repletion, whether selective, is cost-effective in preventing a PJI after a TKA, especially when revision costs exceed $10,636 and the vitamin D-deficiency prevalence is ≥1.1%.
Birinci et al. [27]	Turkey	Overall, 1080 488: vitamin D deficiency and replacement 592: control group—unknown preoperative serum vitamin D status, no supplementation	Male: 219 Female: 861	64.8 (±12.9)	Deficiency: <30 ng/mL	100% in SG (n = 488 patients), but not defined in the control group (n = 592 patients)	Total complications: 4.3% (supplemented) vs. 8.6% (control); *p* = 0.005. Superficial wound infection: 0.2% vs. 2.5%; *p* < 0.001. Postoperative cellulitis: 0% vs. 2.2%; *p* < 0.001. PJI: Similar rates; *p* = 0.23. 90-day mortality: similar; *p* = 0.524. Readmission rate: similar; *p* = 0.683.	Preoperative correction reduced the rates of superficial wound infections and cellulitis, and halved the overall complication rates (4.3% vs. 8.6%) within 90 days, while the PJI, mortality, and readmission remained unchanged.
Kong et al. [28]	Republic of Korea	142,147 undergoing TKA; 28,403 were calcium and vitamin D users and 113,744 were never users	Male: 33,049 Female: 109,098	68.8			Association between calcium and vitamin D use and the revision rates of primary TKA.	Calcium and vitamin D combination use for more than 1 year was associated with reduced revision risks in both patients with a PJI (aHR: 0.63, 95% CI: 0.42–0.95) and patients without an infection (aHR: 0.70, 95% CI: 0.54–0.91). Implant survival was significantly improved in calcium and vitamin D combination users for more than 1 year compared with never-users (log-rank *p* < 0.001).
Zajonz et al. [29]	Germany	240 patients SG (patients with PPI): 80 CG I (patients with primary implants): 80 CG II (patients undergoing aseptic revision): 80	SG: male, 39; female, 41 CG I: male, 39; female, 41 CG II: male, 35; female, 45	SG: 74 (35–92) CG I: 71 (39–87) CG II: 70 (40–85)	Deficiency: <20 ng/mL Insufficiency: 20–29 ng/mL Sufficient: 30–100 ng/mL	SG: 46% CG I: 57% CG II: 47.5%	PJI.	Vitamin D deficiency is common among patients with a PJI, especially in those with acute infections. Patients with an acute PJI had significantly lower levels of 25(OH)D3 compared to patients with chronic PJI (mean: 21.6 ng/mL).
Liu et al. [30]	USA	290 [120 undergoing TKA; 170 isolated distal Darius frac-ture (DRF)]	Male, 58; female, 62 [undergoing TKA] Male, 9; female, 161 [isolated DRF]	Mean: 64.8 [undergoing TKA]; 65.7 [isolated DRF]	<20 ng/mL, deficient; <30 ng/mL, insufficient	TKA: 33.3% deficient, 66.7% insufficient; DRF: 22.9% deficient, 37.1% insufficient	Fragility fractures, DRF, periprosthetic fractures, periprosthetic stem subsidence, aseptic loosening, increased risk of infection, SSI, PJI, septic complications, increased morbidity, increased mortality, DVT, cerebrovascular accidents, postoperative cognitive dysfunction, poorer functional outcomes.	Mean 25OHD levels were significantly lower in DRF (17.5 ng/mL) compared to TKA patients (22.6 ng/mL; *p* < 0.00001). Deficiency: 33.3% of TKA patients and 22.9% of DRF, DRF cohort showing a significantly higher major osteoporotic fracture risk based on FRAX scores (19.7 vs. 7.9; *p* < 0.00001).
Horas et al. [31]	Germany	249 [191 without supplementation; 58 with supplementation]	Male: 107 (43%) Female: 142 (57%)	Mean: 68.29	<20 ng/mL	81% (155/191) without supplementation (53% deficient, 28% insufficient)	Revision arthroplasty due to: periprosthetic infection: 34%; aseptic loosening of another complication: 64%; periprosthetic fractures: 2%.	High rate of Vitamin D deficiency in patients scheduled for an revision TJA. Low vitamin D levels are associated with poorer outcomes in TJA patients, possibly leading to an revision.
Maier et al. [32]	Germany	109: primary arthroplasty; 50: PJI; 31: aseptic loosening	Primary arthroplasty: male, 47; female, 62 PJI: male, 24; female, 26 Aseptic loosening: male, 13; female, 18	65 (± 9.2): primary arthroplasty; 68 (±16): PJI; 68.4 (± 8.6): aseptic loosening	<20 ng/mL	Primary arthroplasty: 64%; PJI: 86%; aseptic loosening: 52%	PJI; aseptic loosening of the prosthesis.	Vitamin D deficiency was significantly severe in patients with: PJI (mean: 13.29 ng/mL); aseptic loosening (*p* < 0.001); primary arthroplasty (*p* < 0.001). High rate of vitamin D deficiency in patients scheduled for an revision TJA.
Hegde et al. [33]	USA	6593 Deficient: 868 Sufficient: 5725	Vit. D-deficient: male, 197; female, 671 Vit. D-sufficient: male, 1402; female, 4323	Not specified	<20 ng/mL	13.2% (of TKA patients tested pre-op)	Postoperative complications: prosthesis explantation (OR: 2.97) (<0.001); SSI (OR: 1.76) (*p* = 0.001); stiffness (MUA) (OR: 1.69) (*p* < 0.001); DVT (OR: 1.80) (*p* < 0.001); myocardial infarction (OR: 2.11) (*p* < 0.001); cerebrovascular accident (OR: 1.73) (*p* = 0.006); tibial/femoral revision (at 3 months) (OR: 1.47) (*p* = 0.226); tibial/femoral revision (at 1 year) (OR: 1.26) (*p* = 0.276).	Vitamin D deficiency is an independent risk factor for both surgical and medical complications following a TKA.
Pilc et al. [34]	USA	8780 Deficient: 4390 Sufficient: 4390	Male: 4312 Female: 5684	69.1	<30 ng/mL	50% (matched cohort)	Periprosthetic fracture in deficient group was 0.3% and in sufficient group was 0.5% (*p* = 0.22). Risks of revision (*p* = 0.57), PJI (*p* = 0.20), aseptic loosening (*p* = 0.80).	No difference in the incidence of vitamin D deficiency between patients who sustained a Periprosthetic fracture, revision arthroplasty, PJI, or aseptic loosening compared to matched controls after a TKA.

## Data Availability

The data presented in this study are available on request from the corresponding author.

## Abbreviations

The following abbreviations are used in this manuscript:

DRF	Distal Radius Fracture
DVT	Deep Vein Thrombosis
KSS	Knee Society Score
OA	Osteoarthritis
PROMs	Patient-Reported Outcome Measures
TJA	Total Joint Arthroplasty
TKA	Total Knee Arthroplasty
BMI	Body Mass Index
PJI	Periprosthetic Joint Infection
SSI	Surgical Site Infection
TUGT	Timed Up and Go Test
WOMAC	Western Ontario and McMaster Universities Osteoarthritis Index
